# 6-[(2-Chloro­pyridin-5-ylmeth­yl)(eth­yl)­azan­yl]-4-(2-fluoro­phen­yl)-1-methyl-5-nitro-1,2,3,4-tetra­hydro­pyridin-2-one

**DOI:** 10.1107/S1600536812005776

**Published:** 2012-02-17

**Authors:** Chuan-Wen Sun, Jing Wang, Ying Wu

**Affiliations:** a Department of Chemistry, College of Life and Environmental Science, Shanghai Normal University, Shanghai 200234, People’s Republic of China

## Abstract

In the title compound, C_20_H_20_ClFN_4_O_3_, the tetra­hydro­pyridone ring adopts a skew boat conformation. The dihedral angle between the mean planes of the benzene and pyridine rings is 80.7 (3)°. In the crystal, weak C—H⋯O inter­actions are observed.

## Related literature

For general background to neonicotinoid compounds and their application as insecticides, see: Jeschke & Nauen (2008 ▶); Kagabu & Matsuno (1997 ▶); Ohno *et al.* (2009 ▶); Shao *et al.* (2008 ▶); Tian *et al.* (2007 ▶); Tomizawa & Casida (2009 ▶). For the synthesis of the title compound, see: Zhang *et al.* (2010 ▶). For puckering parameters, see Cremer & Pople (1975 ▶). 
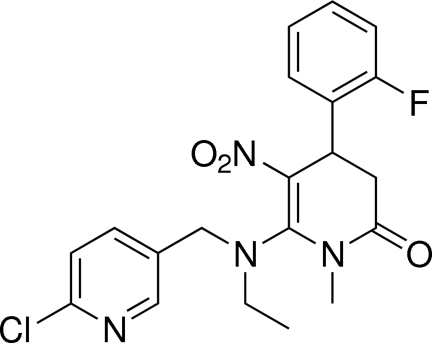


## Experimental

### 

#### Crystal data

C_20_H_20_ClFN_4_O_3_
*M* *_r_* = 418.85
Triclinic, 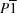

*a* = 6.750 (2) Å
*b* = 8.262 (3) Å
*c* = 17.853 (6) Å
α = 94.981 (6)°
β = 91.302 (7)°
γ = 100.593 (7)°
*V* = 974.2 (6) Å^3^
*Z* = 2
Mo *K*α radiation
μ = 0.24 mm^−1^
*T* = 298 K
0.23 × 0.20 × 0.20 mm

#### Data collection

Bruker SMART CCD area-detector diffractometer
Absorption correction: multi-scan (*SADABS*; Sheldrick, 2003 ▶) *T*_min_ = 0.938, *T*_max_ = 0.954
5691 measured reflections
3391 independent reflections
1943 reflections with *I* > 2σ(*I*)
*R*_int_ = 0.073

#### Refinement

*R*[*F* ^2^ > 2σ(*F* ^2^)] = 0.070
*wR*(*F* ^2^) = 0.151
*S* = 0.96
3391 reflections
264 parameters
H-atom parameters constrained
Δρ_max_ = 0.23 e Å^−3^
Δρ_min_ = −0.25 e Å^−3^

### 

Data collection: *SMART* (Bruker, 2001 ▶); cell refinement: *SMART*; data reduction: *SAINT* (Bruker, 2001 ▶); program(s) used to solve structure: *SHELXS97* (Sheldrick, 2008 ▶); program(s) used to refine structure: *SHELXL97* (Sheldrick, 2008 ▶); molecular graphics: *SHELXTL* (Sheldrick, 2008 ▶); software used to prepare material for publication: *SHELXTL*.

## Supplementary Material

Crystal structure: contains datablock(s) I, global. DOI: 10.1107/S1600536812005776/jj2119sup1.cif

Structure factors: contains datablock(s) I. DOI: 10.1107/S1600536812005776/jj2119Isup2.hkl

Supplementary material file. DOI: 10.1107/S1600536812005776/jj2119Isup3.mol

Supplementary material file. DOI: 10.1107/S1600536812005776/jj2119Isup4.cml

Additional supplementary materials:  crystallographic information; 3D view; checkCIF report

## Figures and Tables

**Table 1:** Hydrogen-bond geometry (Å, °)

*D*—H⋯*A*	*D*—H	H⋯*A*	*D*⋯*A*	*D*—H⋯*A*
C8—H8*C*⋯O1^i^	0.96	2.46	3.383 (5)	161
C6—H6*A*⋯O3^ii^	0.97	2.57	3.506 (5)	161
C3—H3⋯O1	0.93	2.53	3.320 (5)	143
C2—H2⋯O3^iii^	0.93	2.46	3.344 (5)	159

Symmetry codes: (i) 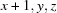; (ii) 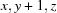; (iii) 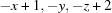.

**Fig. 1.**
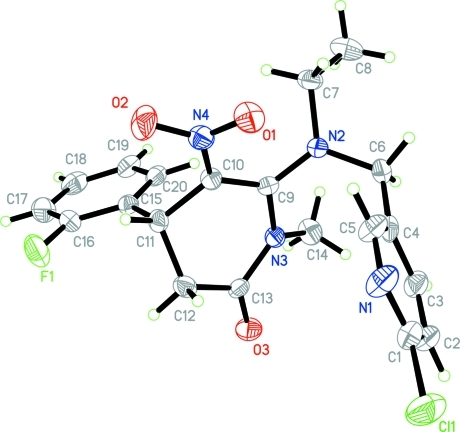
The molecular structure of (I), showing the atom-numbering scheme. Displacement ellipsoids are drawn at the 30% probability level.

**Fig. 2.**
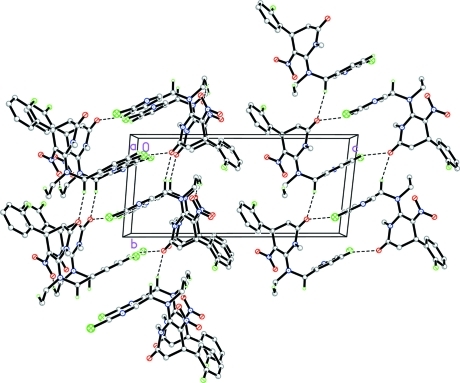
Packing of the title compound, (I), viewed along the a axis. Weak C2—H2···O3, C8—H8C···O1 and C6—H6A···O3 intermolecular interactions are shown as dashed lines. H atoms not involved in hydrogen bonding have been omitted for clarity.

## supplementary crystallographic information

### Comment

In recent years, neonicotinoid insecticide compounds have rapidly grown and become a new chemical class of insecticides because of their novel structure and mode of action (Jeschke & Nauen,2008; Kagabu & Matsuno,1997; Ohno *et al.*, 2009; Shao *et al.*, 2008; Tian *et al.*, 2007; Tomizawa & Casida, 2009). We report here the synthesis and crystal structure of one of these compounds, C_20_H_20_ClFN_4_O_3_, the title compound, (I).

In (I) the tetrahydropyridone ring adopts a skew boat conformation with puckering paramers (Cremer & Pople, 1975) Q, θ and φ of 0.526 (4)Å, 113.2 (4)° and 22.0 (4)°) and a N2/C9/C10/N4 dihedral angle of -21.6 (6)° (Fig.1). In the tetrahydropyridone moiety, C11—C10, C12—C11 and C13—C12 bond lengths (1.499 (5) Å, 1.527 (5) Å and 1.503 (5) Å) are slightly shorter than normal. The C13—N3, C9—N3, and C10—N4 bond lengths (1.391 (4)Å, 1.417 (4)Å, and 1.422 (4)Å, respectively) are slightly shorter than normal while the C9═C10 bond length (1.360 (4)Å) is slightly longer than normal. The dihedral angle between the mean planes of the benzene and pryidine rings is 80.7 (3)°. In the crystal structure weak C2—H2···O3, C8—H8C···O1 and C6—H6A···O3 intermolecular interactions are observed which may influence crystal packing (Fig.2).

### Experimental

In the preparation of the title compound, a solution of 2-fluorobenzaldehyde (15 mmol), Meldrum's acid (15 mmol) in ethanol (30 ml), with piperidine (0.1 mmol) used as catalyst, was added dropwise and the solution was stirred at room temperature for 2 h. Nitenpyram (2.75 g, 10 mmol) was added to the reaction mixture, heated to 65 °C for 6 h and then cooled to room temperature. The reaction mixture was concentrated under reduced pressure and treated with 20 ml of water whereby the solution was extracted three times with CH_2_Cl_2_, and the combined extracts were dried over MgSO_4_. The organic phase was evaporated under reduced pressure and the crude product was subjected to flash chromatography on silica gel, eluting with ethyl acetate /petroleum ether to afford pure yellow crystals (yield 81%). Anal. calcd. for C_20_H_20_ClFN_4_O_3_ C 57.33, H 4.80, N 13.36% found, C 57.35, H 4.81, N 13.38%.

### Refinement

All H atoms bonded to C were positioned geometrically [C—H = 0.93 Å (aromatic), 0.97 Å (methylene) and 0.96Å (methyl)] and refined in the riding mode with [*U*_iso_(H) = 1.2*U*_eq_(aromatic and methylene C) and 1.5*U*_eq_(methyl C)].

### Crystal data

**Table:**

C_20_H_20_ClFN_4_O_3_	*Z* = 2
*M**_r_* = 418.85	*F*(000) = 436
Triclinic, *P*1	*D*_x_ = 1.428 Mg m^−^^3^
Hall symbol: -P 1	Mo *K*α radiation, λ = 0.71073 Å
*a* = 6.750 (2) Å	Cell parameters from 906 reflections
*b* = 8.262 (3) Å	θ = 2.3–19.9°
*c* = 17.853 (6) Å	µ = 0.24 mm^−^^1^
α = 94.981 (6)°	*T* = 298 K
β = 91.302 (7)°	Block, yellow
γ = 100.593 (7)°	0.23 × 0.20 × 0.20 mm
*V* = 974.2 (6) Å^3^	

### Data collection

**Table:**

Bruker SMART CCD area-detector diffractometer	3391 independent reflections
Radiation source: fine-focus sealed tube	1943 reflections with *I* > 2σ(*I*)
Graphite monochromator	*R*_int_ = 0.073
phi and ω scans	θ_max_ = 25.0°, θ_min_ = 2.3°
Absorption correction: multi-scan (*SADABS*; Sheldrick, 2003)	*h* = −7→8
*T*_min_ = 0.938, *T*_max_ = 0.954	*k* = −9→9
5691 measured reflections	*l* = −21→16

### Refinement

**Table:**

Refinement on *F*^2^	Primary atom site location: structure-invariant direct methods
Least-squares matrix: full	Secondary atom site location: difference Fourier map
*R*[*F*^2^ > 2σ(*F*^2^)] = 0.070	Hydrogen site location: inferred from neighbouring sites
*wR*(*F*^2^) = 0.151	H-atom parameters constrained
*S* = 0.96	*w* = 1/[σ^2^(*F*_o_^2^) + (0.0497*P*)^2^] where *P* = (*F*_o_^2^ + 2*F*_c_^2^)/3
3391 reflections	(Δ/σ)_max_ < 0.001
264 parameters	Δρ_max_ = 0.23 e Å^−^^3^
0 restraints	Δρ_min_ = −0.25 e Å^−^^3^

### Special details

**Table:**

Geometry. All e.s.d.'s (except the e.s.d. in the dihedral angle between two l.s. planes) are estimated using the full covariance matrix. The cell e.s.d.'s are taken into account individually in the estimation of e.s.d.'s in distances, angles and torsion angles; correlations between e.s.d.'s in cell parameters are only used when they are defined by crystal symmetry. An approximate (isotropic) treatment of cell e.s.d.'s is used for estimating e.s.d.'s involving l.s. planes.
Refinement. Refinement of *F*^2^ against ALL reflections. The weighted *R*-factor *wR* and goodness of fit *S* are based on *F*^2^, conventional *R*-factors *R* are based on *F*, with *F* set to zero for negative *F*^2^. The threshold expression of *F*^2^ > σ(*F*^2^) is used only for calculating *R*-factors(gt) *etc*. and is not relevant to the choice of reflections for refinement. *R*-factors based on *F*^2^ are statistically about twice as large as those based on *F*, and *R*- factors based on ALL data will be even larger.

### Fractional atomic coordinates and isotropic or equivalent isotropic displacement parameters (Å^2^)

**Table:**

	*x*	*y*	*z*	*U*_iso_*/*U*_eq_	
Cl1	−0.0482 (2)	0.19595 (18)	1.06337 (7)	0.0963 (5)	
F1	0.0556 (4)	−0.3141 (3)	0.57751 (14)	0.0768 (8)	
O1	0.1183 (4)	0.3810 (3)	0.70839 (17)	0.0579 (8)	
O2	0.0164 (4)	0.1954 (4)	0.61628 (17)	0.0745 (10)	
O3	0.4797 (4)	−0.1603 (3)	0.82425 (15)	0.0613 (8)	
N1	0.0698 (5)	0.3252 (5)	0.9407 (2)	0.0677 (11)	
N2	0.5075 (4)	0.3694 (3)	0.76441 (15)	0.0358 (7)	
N3	0.5073 (4)	0.0931 (3)	0.78309 (15)	0.0367 (7)	
N4	0.1287 (5)	0.2474 (4)	0.67181 (19)	0.0457 (8)	
C1	0.1333 (7)	0.2582 (5)	0.9985 (2)	0.0579 (12)	
C2	0.3272 (8)	0.2367 (5)	1.0112 (2)	0.0620 (13)	
H2	0.3632	0.1866	1.0528	0.074*	
C3	0.2101 (7)	0.3769 (5)	0.8917 (2)	0.0595 (12)	
H3	0.1690	0.4245	0.8501	0.071*	
C4	0.4110 (6)	0.3647 (4)	0.8982 (2)	0.0431 (10)	
C5	0.4664 (6)	0.2921 (5)	0.9599 (2)	0.0539 (11)	
H5	0.5998	0.2804	0.9669	0.065*	
C6	0.5569 (6)	0.4366 (4)	0.8427 (2)	0.0475 (11)	
H6A	0.5631	0.5553	0.8461	0.057*	
H6B	0.6899	0.4176	0.8567	0.057*	
C7	0.5241 (6)	0.4907 (4)	0.7083 (2)	0.0468 (10)	
H7A	0.4291	0.5638	0.7190	0.056*	
H7B	0.4883	0.4331	0.6588	0.056*	
C8	0.7339 (6)	0.5922 (5)	0.7079 (3)	0.0664 (13)	
H8A	0.7682	0.6524	0.7563	0.100*	
H8B	0.7388	0.6686	0.6701	0.100*	
H8C	0.8283	0.5204	0.6970	0.100*	
C9	0.4199 (5)	0.2097 (4)	0.74628 (18)	0.0333 (9)	
C10	0.2653 (5)	0.1481 (4)	0.6955 (2)	0.0360 (9)	
C11	0.2062 (5)	−0.0340 (4)	0.6735 (2)	0.0387 (9)	
H11	0.0674	−0.0550	0.6527	0.046*	
C12	0.2044 (5)	−0.1165 (4)	0.7469 (2)	0.0444 (10)	
H12A	0.0999	−0.0843	0.7782	0.053*	
H12B	0.1738	−0.2357	0.7359	0.053*	
C13	0.4050 (6)	−0.0674 (5)	0.78885 (19)	0.0401 (9)	
C14	0.7233 (5)	0.1245 (5)	0.8024 (2)	0.0539 (11)	
H14A	0.7442	0.1457	0.8560	0.081*	
H14B	0.7887	0.2190	0.7787	0.081*	
H14C	0.7789	0.0297	0.7852	0.081*	
C15	0.3356 (5)	−0.1057 (4)	0.6151 (2)	0.0371 (9)	
C16	0.2525 (6)	−0.2468 (5)	0.5697 (2)	0.0491 (11)	
C17	0.3578 (7)	−0.3229 (5)	0.5167 (2)	0.0621 (13)	
H17	0.2958	−0.4175	0.4870	0.074*	
C18	0.5569 (7)	−0.2557 (5)	0.5088 (2)	0.0590 (12)	
H18	0.6323	−0.3060	0.4740	0.071*	
C19	0.6450 (6)	−0.1144 (5)	0.5521 (2)	0.0500 (11)	
H19	0.7796	−0.0685	0.5462	0.060*	
C20	0.5345 (6)	−0.0401 (5)	0.6044 (2)	0.0418 (10)	
H20	0.5957	0.0563	0.6330	0.050*	

### Atomic displacement parameters (Å^2^)

**Table:**

	*U*^11^	*U*^22^	*U*^33^	*U*^12^	*U*^13^	*U*^23^
Cl1	0.1069 (11)	0.1166 (12)	0.0622 (9)	0.0018 (9)	0.0239 (8)	0.0258 (8)
F1	0.0722 (17)	0.0626 (16)	0.0813 (18)	−0.0162 (13)	0.0080 (14)	−0.0141 (14)
O1	0.0526 (17)	0.0505 (18)	0.077 (2)	0.0280 (15)	−0.0045 (15)	0.0027 (16)
O2	0.074 (2)	0.072 (2)	0.077 (2)	0.0232 (17)	−0.0420 (18)	−0.0028 (17)
O3	0.087 (2)	0.0429 (17)	0.0585 (19)	0.0198 (16)	−0.0108 (16)	0.0156 (15)
N1	0.062 (2)	0.098 (3)	0.044 (2)	0.014 (2)	0.0000 (19)	0.013 (2)
N2	0.0406 (18)	0.0324 (17)	0.0344 (17)	0.0059 (14)	0.0014 (14)	0.0049 (14)
N3	0.0360 (17)	0.0358 (17)	0.0382 (18)	0.0071 (14)	−0.0067 (14)	0.0046 (14)
N4	0.0395 (19)	0.045 (2)	0.054 (2)	0.0081 (16)	−0.0040 (17)	0.0101 (17)
C1	0.073 (3)	0.059 (3)	0.038 (3)	0.006 (2)	0.003 (2)	−0.001 (2)
C2	0.089 (4)	0.059 (3)	0.038 (3)	0.010 (3)	−0.007 (2)	0.010 (2)
C3	0.067 (3)	0.077 (3)	0.037 (2)	0.015 (3)	−0.002 (2)	0.013 (2)
C4	0.050 (3)	0.040 (2)	0.036 (2)	0.008 (2)	−0.0077 (19)	−0.0111 (18)
C5	0.064 (3)	0.056 (3)	0.042 (3)	0.015 (2)	−0.012 (2)	0.004 (2)
C6	0.055 (3)	0.037 (2)	0.046 (2)	0.0004 (19)	−0.008 (2)	−0.0034 (18)
C7	0.056 (3)	0.037 (2)	0.050 (2)	0.0093 (19)	0.004 (2)	0.0132 (19)
C8	0.059 (3)	0.054 (3)	0.089 (4)	0.007 (2)	0.017 (3)	0.028 (3)
C9	0.036 (2)	0.035 (2)	0.031 (2)	0.0089 (17)	0.0030 (17)	0.0075 (16)
C10	0.033 (2)	0.035 (2)	0.041 (2)	0.0104 (17)	−0.0037 (17)	0.0038 (17)
C11	0.032 (2)	0.040 (2)	0.042 (2)	0.0021 (17)	−0.0060 (17)	0.0020 (18)
C12	0.046 (2)	0.040 (2)	0.045 (2)	0.0019 (18)	0.0107 (19)	0.0051 (18)
C13	0.056 (3)	0.039 (2)	0.026 (2)	0.012 (2)	0.0009 (18)	0.0049 (17)
C14	0.043 (2)	0.052 (3)	0.068 (3)	0.014 (2)	−0.010 (2)	0.003 (2)
C15	0.042 (2)	0.035 (2)	0.035 (2)	0.0100 (18)	−0.0018 (17)	0.0062 (17)
C16	0.054 (3)	0.041 (2)	0.048 (3)	−0.003 (2)	−0.001 (2)	0.006 (2)
C17	0.089 (4)	0.046 (3)	0.049 (3)	0.013 (3)	0.011 (3)	−0.006 (2)
C18	0.076 (3)	0.065 (3)	0.044 (3)	0.031 (3)	0.009 (2)	0.010 (2)
C19	0.054 (3)	0.064 (3)	0.037 (2)	0.022 (2)	0.001 (2)	0.009 (2)
C20	0.049 (2)	0.046 (2)	0.032 (2)	0.011 (2)	−0.0010 (18)	0.0057 (18)

### Geometric parameters (Å, º)

**Table:**

Cl1—C1	1.744 (4)	C7—H7B	0.9700
F1—C16	1.358 (4)	C8—H8A	0.9600
O1—N4	1.247 (4)	C8—H8B	0.9600
O2—N4	1.231 (4)	C8—H8C	0.9600
O3—C13	1.206 (4)	C9—C10	1.360 (4)
N1—C1	1.312 (5)	C10—C11	1.499 (5)
N1—C3	1.342 (5)	C11—C15	1.526 (5)
N2—C9	1.352 (4)	C11—C12	1.527 (5)
N2—C6	1.467 (4)	C11—H11	0.9800
N2—C7	1.469 (4)	C12—C13	1.503 (5)
N3—C13	1.391 (4)	C12—H12A	0.9700
N3—C9	1.417 (4)	C12—H12B	0.9700
N3—C14	1.461 (4)	C14—H14A	0.9600
N4—C10	1.422 (4)	C14—H14B	0.9600
C1—C2	1.370 (6)	C14—H14C	0.9600
C2—C5	1.373 (5)	C15—C20	1.376 (5)
C2—H2	0.9300	C15—C16	1.381 (5)
C3—C4	1.381 (5)	C16—C17	1.373 (5)
C3—H3	0.9300	C17—C18	1.372 (5)
C4—C5	1.374 (5)	C17—H17	0.9300
C4—C6	1.495 (5)	C18—C19	1.371 (5)
C5—H5	0.9300	C18—H18	0.9300
C6—H6A	0.9700	C19—C20	1.381 (5)
C6—H6B	0.9700	C19—H19	0.9300
C7—C8	1.508 (5)	C20—H20	0.9300
C7—H7A	0.9700		
			
C1—N1—C3	115.8 (4)	N2—C9—N3	115.0 (3)
C9—N2—C6	121.8 (3)	C10—C9—N3	116.8 (3)
C9—N2—C7	121.2 (3)	C9—C10—N4	121.7 (3)
C6—N2—C7	116.1 (3)	C9—C10—C11	121.2 (3)
C13—N3—C9	122.3 (3)	N4—C10—C11	116.0 (3)
C13—N3—C14	115.9 (3)	C10—C11—C15	115.7 (3)
C9—N3—C14	120.6 (3)	C10—C11—C12	105.8 (3)
O2—N4—O1	121.0 (3)	C15—C11—C12	112.4 (3)
O2—N4—C10	118.5 (3)	C10—C11—H11	107.5
O1—N4—C10	120.4 (3)	C15—C11—H11	107.5
N1—C1—C2	125.1 (4)	C12—C11—H11	107.5
N1—C1—Cl1	115.6 (4)	C13—C12—C11	110.8 (3)
C2—C1—Cl1	119.3 (4)	C13—C12—H12A	109.5
C1—C2—C5	117.3 (4)	C11—C12—H12A	109.5
C1—C2—H2	121.4	C13—C12—H12B	109.5
C5—C2—H2	121.4	C11—C12—H12B	109.5
N1—C3—C4	125.0 (4)	H12A—C12—H12B	108.1
N1—C3—H3	117.5	O3—C13—N3	120.4 (3)
C4—C3—H3	117.5	O3—C13—C12	123.4 (3)
C5—C4—C3	116.0 (4)	N3—C13—C12	116.2 (3)
C5—C4—C6	123.5 (4)	N3—C14—H14A	109.5
C3—C4—C6	120.3 (4)	N3—C14—H14B	109.5
C2—C5—C4	120.8 (4)	H14A—C14—H14B	109.5
C2—C5—H5	119.6	N3—C14—H14C	109.5
C4—C5—H5	119.6	H14A—C14—H14C	109.5
N2—C6—C4	114.6 (3)	H14B—C14—H14C	109.5
N2—C6—H6A	108.6	C20—C15—C16	116.4 (4)
C4—C6—H6A	108.6	C20—C15—C11	124.4 (3)
N2—C6—H6B	108.6	C16—C15—C11	119.2 (3)
C4—C6—H6B	108.6	F1—C16—C17	118.1 (4)
H6A—C6—H6B	107.6	F1—C16—C15	118.4 (4)
N2—C7—C8	112.1 (3)	C17—C16—C15	123.6 (4)
N2—C7—H7A	109.2	C18—C17—C16	118.3 (4)
C8—C7—H7A	109.2	C18—C17—H17	120.9
N2—C7—H7B	109.2	C16—C17—H17	120.9
C8—C7—H7B	109.2	C19—C18—C17	120.1 (4)
H7A—C7—H7B	107.9	C19—C18—H18	119.9
C7—C8—H8A	109.5	C17—C18—H18	119.9
C7—C8—H8B	109.5	C18—C19—C20	120.2 (4)
H8A—C8—H8B	109.5	C18—C19—H19	119.9
C7—C8—H8C	109.5	C20—C19—H19	119.9
H8A—C8—H8C	109.5	C15—C20—C19	121.4 (4)
H8B—C8—H8C	109.5	C15—C20—H20	119.3
N2—C9—C10	128.1 (3)	C19—C20—H20	119.3
			
C3—N1—C1—C2	−0.7 (7)	O2—N4—C10—C11	−24.8 (5)
C3—N1—C1—Cl1	178.5 (3)	O1—N4—C10—C11	152.5 (3)
N1—C1—C2—C5	1.1 (7)	C9—C10—C11—C15	−80.5 (4)
Cl1—C1—C2—C5	−178.1 (3)	N4—C10—C11—C15	111.0 (3)
C1—N1—C3—C4	−0.2 (7)	C9—C10—C11—C12	44.6 (4)
N1—C3—C4—C5	0.7 (6)	N4—C10—C11—C12	−123.9 (3)
N1—C3—C4—C6	−175.9 (4)	C10—C11—C12—C13	−56.2 (4)
C1—C2—C5—C4	−0.5 (6)	C15—C11—C12—C13	70.9 (4)
C3—C4—C5—C2	−0.3 (6)	C9—N3—C13—O3	−175.8 (3)
C6—C4—C5—C2	176.2 (3)	C14—N3—C13—O3	17.0 (5)
C9—N2—C6—C4	−34.4 (5)	C9—N3—C13—C12	6.1 (5)
C7—N2—C6—C4	134.8 (3)	C14—N3—C13—C12	−161.1 (3)
C5—C4—C6—N2	124.5 (4)	C11—C12—C13—O3	−143.5 (4)
C3—C4—C6—N2	−59.1 (5)	C11—C12—C13—N3	34.5 (4)
C9—N2—C7—C8	−131.8 (4)	C10—C11—C15—C20	27.1 (5)
C6—N2—C7—C8	58.8 (4)	C12—C11—C15—C20	−94.5 (4)
C6—N2—C9—C10	136.3 (4)	C10—C11—C15—C16	−153.7 (3)
C7—N2—C9—C10	−32.4 (5)	C12—C11—C15—C16	84.6 (4)
C6—N2—C9—N3	−46.4 (4)	C20—C15—C16—F1	−179.0 (3)
C7—N2—C9—N3	144.8 (3)	C11—C15—C16—F1	1.8 (5)
C13—N3—C9—N2	160.7 (3)	C20—C15—C16—C17	0.9 (6)
C14—N3—C9—N2	−32.6 (4)	C11—C15—C16—C17	−178.4 (4)
C13—N3—C9—C10	−21.8 (5)	F1—C16—C17—C18	−179.8 (4)
C14—N3—C9—C10	144.9 (3)	C15—C16—C17—C18	0.4 (6)
N2—C9—C10—N4	−21.6 (6)	C16—C17—C18—C19	−1.2 (6)
N3—C9—C10—N4	161.2 (3)	C17—C18—C19—C20	0.7 (6)
N2—C9—C10—C11	170.6 (3)	C16—C15—C20—C19	−1.4 (5)
N3—C9—C10—C11	−6.6 (5)	C11—C15—C20—C19	177.8 (3)
O2—N4—C10—C9	166.7 (3)	C18—C19—C20—C15	0.7 (5)
O1—N4—C10—C9	−16.0 (5)		

### Hydrogen-bond geometry (Å, º)

**Table:**

*D*—H···*A*	*D*—H	H···*A*	*D*···*A*	*D*—H···*A*
C8—H8*C*···O1^i^	0.96	2.46	3.383 (5)	161
C6—H6*A*···O3^ii^	0.97	2.57	3.506 (5)	161
C3—H3···O1	0.93	2.53	3.320 (5)	143
C2—H2···O3^iii^	0.93	2.46	3.344 (5)	159

Symmetry codes: (i) *x*+1, *y*, *z*; (ii) *x*, *y*+1, *z*; (iii) −*x*+1, −*y*, −*z*+2.
